# Forests, Trees, and Micronutrient-Rich Food Consumption in Indonesia

**DOI:** 10.1371/journal.pone.0154139

**Published:** 2016-05-17

**Authors:** Amy Ickowitz, Dominic Rowland, Bronwen Powell, Mohammad Agus Salim, Terry Sunderland

**Affiliations:** 1 Center for International Forestry Research, Jl. CIFOR, Situ Gede, Bogor (Barat) 16115, Indonesia; 2 Department of Geography and African Studies, Pennsylvania State University, University Park, Pennsylvania, United States of America; 3 School of Marine and Environmental Sciences, James Cook University, Cairns, Australia; Indiana University, UNITED STATES

## Abstract

Micronutrient deficiency remains a serious problem in Indonesia with approximately 100 million people, or 40% of the population, suffering from one or more micronutrient deficiencies. In rural areas with poor market access, forests and trees may provide an essential source of nutritious food. This is especially important to understand at a time when forests and other tree-based systems in Indonesia are being lost at unprecedented rates. We use food consumption data from the 2003 Indonesia Demographic Health Survey for children between the ages of one and five years and data on vegetation cover from the Indonesian Ministry of Forestry to examine whether there is a relationship between different tree-dominated land classes and consumption of micronutrient-rich foods across the archipelago. We run our models on the aggregate sample which includes over 3000 observations from 25 provinces across Indonesia as well as on sub-samples from different provinces chosen to represent the different land classes. The results show that different tree-dominated land classes were associated with the dietary quality of people living within them in the provinces where they were dominant. Areas of swidden/agroforestry, natural forest, timber and agricultural tree crop plantations were all associated with more frequent consumption of food groups rich in micronutrients in the areas where these were important land classes. The swidden/agroforestry land class was the landscape associated with more frequent consumption of the largest number of micronutrient rich food groups. Further research needs to be done to establish what the mechanisms are that underlie these associations. Swidden cultivation in is often viewed as a backward practice that is an impediment to food security in Indonesia and destructive of the environment. If further research corroborates that swidden farming actually results in better nutrition than the practices that replace it, Indonesian policy makers may need to reconsider their views on this land use.

## Introduction

Global efforts to improve food security and health increasingly acknowledge the importance of a balanced, micronutrient-rich diet. Adequate intake of micronutrient-rich foods is an essential part of sustainable strategies to prevent micronutrient deficiencies. These deficiencies can hinder both the physical and cognitive development of children as well as increase the risk of infection and childhood mortality particularly among the poor in developing countries [1]. Recognition of the need to increase the consumption of micronutrients-rich foods has stimulated calls to make agricultural policies and practice move beyond the historical focus on yield and energy (calorie) availability to also take into account dietary quality [2]. Forests and trees may play an important role in the nutrition-sensitivity of the food system and broader landscape [3, 4].

Forests and trees can contribute to food security and nutrition in at least three ways. First, forests contribute indirectly to food security through the ecosystems services that they provide to agriculture [5–7]. Second, smallholder farming practices that are dependent on trees, such as swidden cultivation and agroforestry, likely result in more diverse diets since they produce a variety foods [8]; and dietary diversity is a strong indicator of dietary quality [9, 10]. Third, people living in or near forests can have direct access unmediated by markets to wild foods from forests [11, 12].

Forest foods including fruits, vegetables, fish, bushmeat, and insects, tend to have high nutritional quality. For example, fruits and vegetables are generally rich in many micronutrients, low in fat and high in fiber, while bushmeat, fish, and insects are excellent sources of bio-available micronutrients [4, 13]. In contrast, agriculture often focuses on the production of a handful of staple crops that, on their own, may not provide a balanced diet with sufficient quantities of micronutrients [14]. Forest foods have been documented to contribute to food security and nutrition in regions and countries all over the world; including many countries in Africa [15], Madagascar [16], Ghana [17], Tanzania [13], South Africa [18], Gabon [19] India [20], Argentina [21], Brazil [22], as well as in Indonesia [12, 23–25].

### Nutrition in Indonesia

Indonesia is facing a multitude of food security and nutrition challenges. Despite considerable progress over the last few decades in reducing child mortality and the prevalence of underweight and stunted children, there remains a long way to go before all Indonesian children are free of malnutrition [26]. Over a single decade, from the period 1993–1997 to 2003–2007, child mortality in Indonesia dropped by 33% from 69 to 44 child deaths per 1,000 live births [26], yet the prevalence of stunting in children under 5 years of age remains high at 25.2% and 39.2%, in urban and rural areas respectively [27]. Currently, 29% of Indonesian households have a caloric intake below the recommended daily allowance [28]. Around 40% of the population (100 million Indonesians) suffer from one or more micronutrient deficiencies—the most common being iron, vitamin A, zinc and iodine [29, 30]. Moreover, as Indonesia has developed, chronic, diet-related diseases, have increased dramatically in association with dietary and lifestyle transitions [31], leading to the ‘double burden’ of simultaneous over and under-nutrition. Maternal and Child Double Burden (MCDB), defined as the coexistence of an overweight mother and stunted child in the same household, has an estimated prevalence of 11% in rural Indonesia [32, 33].

Indonesia has had policies in place to combat micronutrient deficiencies since the mid 1970s [34, 35]. The Ministry of Health (MOH) currently implements supplementation programs for two micronutrients; iron and vitamin A; as well as a fortification program for iodine [36]. The effectiveness of supplementation and fortification programs is beyond dispute. Globally, millions of lives are saved, and enormous reductions in morbidity have been and are being achieved through such programs [37]. In Indonesia, significant reductions in child mortality and morbidity can be attributed in part to the success of micronutrient supplementation programs [26, 38–41]. Unlike supplementation and fortification schemes, food based initiatives have received comparatively little attention in national policies [41, 42]. Addressing the causes of undernutrition: poor diets lacking in calories and micronutrients [43], as well as infection [44], is key to overcoming it in a sustainable fashion [45].

Nationally representative data on food intake are not easily available in Indonesia and dietary patterns vary significantly by geographic region reflecting local availability and cultural differences. For instance, while rice is the primary staple food throughout much of Indonesia, sago remains the staple food in Papua province. Likewise, sources of protein vary from island to island, with plant-based protein such as tofu and tempeh (as well as animal-source foods) dominant in Java, while some eastern Islands in Nusa Tenggara are dependent entirely on fish for protein. Despite the variation in dietary patterns, aggregate figures for the country suggest poor dietary quality is one of the major causes of malnutrition in Indonesia [26, 27, 46–48]. Indonesians consume on average about 160 kg of rice per capita per year [49] with very low quantities of vegetables, fruits and animal source foods [50]. The consumption of micronutrient-rich animal source foods remains low—particularly in poor rural environments—with average consumption of meat and fish at 9.4 and 2.2 kg per capita per year respectively [36].

Despite variation in traditional foods, the nutrition transition—experienced near universally across Indonesia—driven by improved access to income and markets is resulting in increased consumption of processed foods (noodles), oils, fats and sugars across the country. Fewer than 26% on the population adheres to the national guidelines of not consuming more than a quarter of energy from fats and oils [36]. Most staple foods in Indonesia are extremely low in iron, zinc and vitamin A, whereas animal source foods, leafy-green vegetables, and fruits contain higher densities of all three micronutrients[51].

Given low overall consumption of animal source foods, fruit, and vegetables, dietary approaches to relieving the burden of malnutrition may be apposite. Studies have shown that increased consumption and expenditure on animal-source foods, leafy-green vegetables and fruits in Indonesia is directly linked to nutritional status [46, 52, 53]. Our understanding of why people in Indonesia are not eating more fruits, vegetables, and animal source foods, remains limited. One possible explanation is that nutrient-dense foods are generally more costly than grains and tubers [54]. It is also possible that in rural areas where infrastructure is poor, some nutritious foods that are perishable and not locally grown are unlikely to be traded in markets and therefore might simply be unavailable. And finally, social and cultural factors may also be affecting peoples’ dietary choices.

If the lack of infrastructure and availability of food are important constraints, then it is likely that in areas where these foods are more available, we will see higher consumption. Since many forest foods, e.g. fruits, vegetables, and wild meat, tend to be micronutrient-dense, we hypothesize that children living in areas with more forests will consume micronutrient-rich foods more frequently. In addition to natural forests, tree-based agricultural systems such as swidden cultivation and smallholder agroforestry that are widely practiced in forested regions may also be positively associated with dietary quality. Swidden cultivation (also known as shifting cultivation and slash-and-burn agriculture] is comprised of many different practices and techniques, but its main defining feature is that fields are cleared for annual or semi-annual crops, then returned to fallow, and subsequently re-cleared in a cyclic fashion. Forest is often cleared to begin the cycle and the fallows can regenerate into forests before they are re-cleared. The landscapes where swidden is prevalent are often characterized by mosaics of forests, fallows, and fields. Swidden cultivation has long been known for the diversity of crops grown [55,56] as well as the management of fallows for wild foods [57]. In addition to the diversity of staple foods that are often intercropped with legumes and vegetables, the management of fruit and nut trees in both fields and fallows, as well as hunting in both fallows and adjacent secondary forests can result in a diverse and nutrient-rich diet.

Landscapes dominated by natural forests and forest mosaics characteristic of swidden and smallholder agroforestry may provide micronutrient-rich foods as explained, but it is also possible that the conversion of such landscapes into commercial plantations either for timber or other tree cash crops (oil palm, rubber, coffee, etc.) can bring infrastructure, development and cash income to remote areas, enabling local people to buy healthy foods. In trying to understand the relationship between forests and diets in Indonesia, we think it is useful to examine both the contributions of forests and mosaics to diets as well as the competing land uses of commercial agriculture. In this study we investigate the relationship between diet and four different tree-dominated land classes in rural Indonesia–natural forests, swidden/smallholder agroforestry, timber plantations, and agricultural tree crop plantations.

## Methods

### Dietary data

We use food consumption data from the 2003 Indonesian Demographic Health Survey (DHS) for children under five years of age. While these data are quite old, this is the only nationally representative data set with information on individual food consumption at a small enough scale (village level) that includes location information enabling us to combine the data with vegetation data on forests and trees.(The DHS has done more recent surveys, but these do not include GPS coordinates. The Indonesian National Economic Survey is done every two years, but the information collected is only on expenditures and is at the household and not individual level. Also, location information is available only for the district and not the village level). The DHS are nationally representative household surveys, focusing on maternal and child health in developing countries. DHS surveys have been carried out in over 90 countries around the world, using standardized methods and questionnaires. The focus of our study is on the frequency with which children consumed items from various food groups over the seven days preceding data collection. We constrained the study to rural areas and included children between the ages of 13 and 59 months so as to exclude children who were mainly consuming breast milk or formula. We use data for only one child per household since there is likely to be correlation of food consumption among children in the same household. Overall, 3,063 observations from 25 provinces were included in our analysis.

The Indonesian DHS includes information on the number of days in the last week that a child consumed from various food groups. We focus our analysis on foods from the following food groups since they tend to be high in micronutrients: vitamin A rich fruits (mango, papaya, durian, jackfruit), leafy-green vegetables (spinach, cassava leaves, etc.), vitamin A rich vegetables (pumpkin, sweet potato, yams, carrots), ‘other’ fruits and vegetables (apples, bananas, avocados, tomatoes, etc.), legumes (lentils, beans, peanuts, soybeans, tofu, tempeh), and animal source foods (meat, poultry, fish, and eggs)[10].

### Spatial data

The main hypothesis that we test is whether there is an association between forests (and other tree-dominated landscapes) and the consumption of micronutrient-rich foods in Indonesia. Since the DHS includes longitude/latitude for each community included in the survey, we were able to join the dataset with 2003 data on vegetation classes from the Indonesian Ministry of Forestry (MOF) [58]. The MOF classifies land use based on Landsat satellite imagery at 30 meters resolution; we focus our analysis on tree-dominated land classes and include the following categories: natural forest (which includes primary forest, secondary forest, and swamp forest), agricultural plantations (palm oil, rubber, coffee, etc.), timber plantations (teak, *Acacia*, etc.) derived from the MOF classifications. These categories are relatively straightforward. We also create a land use category of swidden/agroforestry based on the MOF category of ‘upland farming mixed with bush’. The description that the MOF uses for this class is: “*An entire scene of upland farming and plantation*, *which muddles up with shrub*, *bush*, *and logged forest*. *It is frequently found in shifting cultivation area*, *and karst land planting rotation*. *This classification also includes mixed plantation*, *dominated by tree crops (plantation trees) in between the shrub*.*”* [59] This category is by no means definitive, but since there are no aggregate statistics for land area used for swidden and agroforestry for Indonesia, we use this information under the assumption that there will be some ‘signal’ in the data even though there will likely be some measurement error.

**Fig 1:**
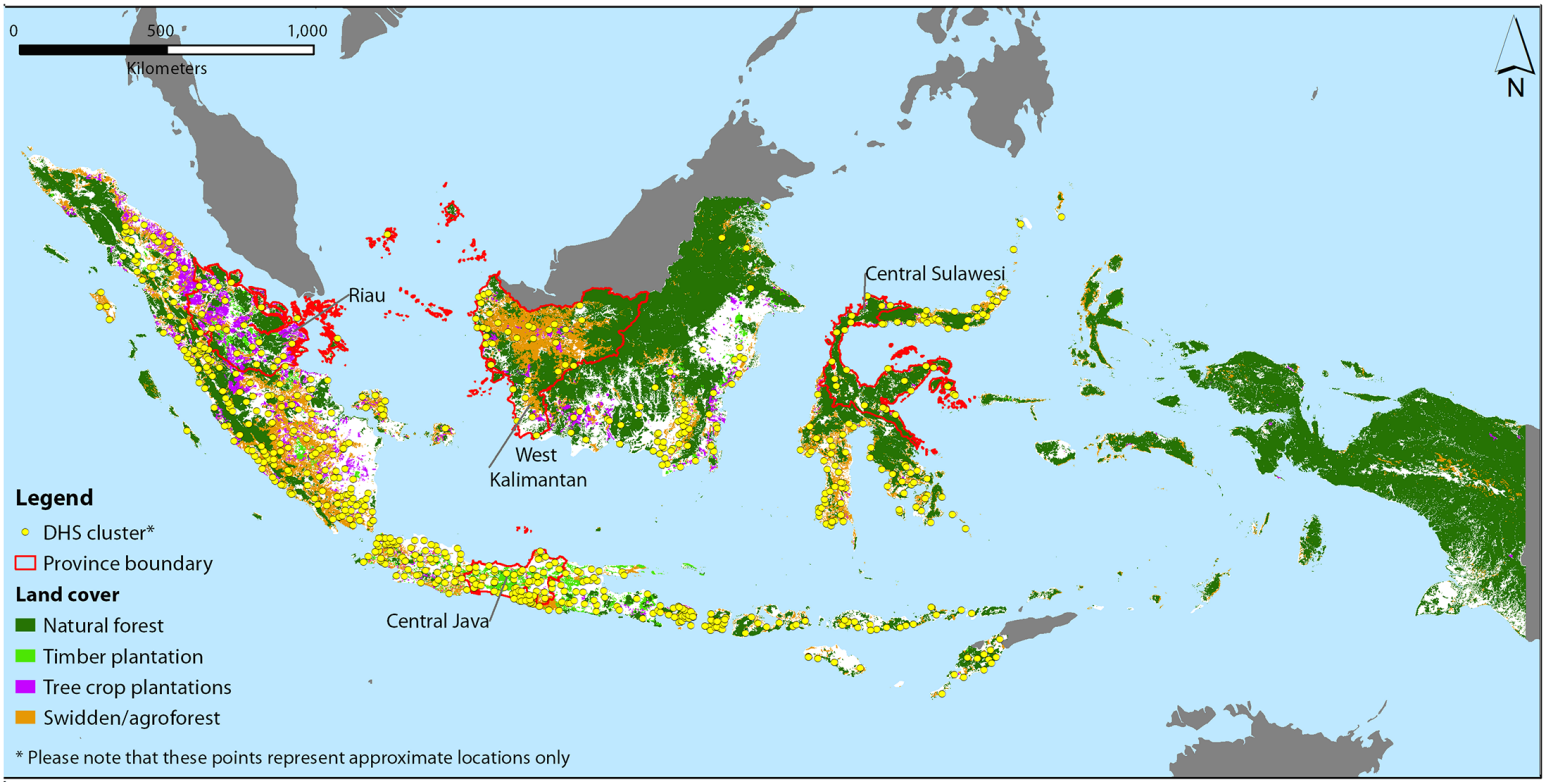
Indonesia Map. The approximate location of the DHS communities used in the study as well as the different tree-dominated land classes used in the analysis from Indonesian Ministry of Forestry.

We calculated the average area for each of the different classes for a 5km radius around the village coordinates provided by the DHS. The DHS does not report exact coordinates for villages, but displaces 99% by up to 5 km (and 1% by up to 10%) to protect anonymity of communities. Fig 1 shows the approximate location of the DHS communities used in the study as well as the different vegetation classes used in the analysis.

### Independent Variables

Several individual level characteristics of the child may affect consumption patterns and therefore were controlled for in the model. Older children tend to eat more foods both in quantity and diversity so we control for both the age of the child as well as age squared. We excluded children under thirteen months from the study, as there is a high probability that they would be getting a substantial percentage of their calories from breastfeeding. However, 41% of the children in the sample were still breastfeeding to some degree, so we also controlled for this effect by including a dummy variable equal to one if the child was breastfeeding at the time of the survey. Since it is possible that there might be cultural norms about feeding boys and girls different foods, we included a dummy equal to one if a child was male.

Various household characteristics are known to affect the consumption of nutritious foods. The effect of the parents’ education on a child’s nutrition has been well documented so we include the number of years of the father’s education [60]. Wealthier households may be able to afford to buy more food and food that may be of higher quality. Most food groups show some degree of income elasticity in Indonesia, with animal source foods showing higher income elasticities than other food groups [61]. Income data is not collected by DHS surveys, but the DHS includes an asset-based wealth score which is constructed by principle component analysis based on the methodology developed by Filmer and Prichett [62]. Although assets do not equate to income available for food, they represent a good proxy for the relative wealth of a household over time, are less susceptible to measurement error and have been shown to be strong predictors of child nutrition outcomes [63]. The religion of the household also may affect dietary patterns. The most obvious example is that Muslims are forbidden to consume certain types of meat including pork; it is especially important to address this issue for this study since wild pigs are an important source of meat for many people living near forests in Indonesia. We, therefore, also include a dummy variable equal to one if the head of household is Muslim.

Geographical factors such as aridity and elevation can affect the type of agricultural practices as well as the types of crops grown which in turn can affect which wild foods are available. Proximity to the coast is also likely to be important for both access to markets as well as for consumption of fish and other seafood. Since the DHS does not distinguish between fish (or any of the animal source foods), we control for proximity to the sea by including the distance from the village to the coast as a variable in our model. Rivers can be important in some regions for fish so we also include distance to closest to river. The level of market integration is an important concern; households with poor access to markets are more dependent on self-produced food and are often subject to higher market prices for purchased food. In contrast, households with good access to markets may be more dependent on market foods, making them more susceptible to food price fluctuations [64]. We use distance to nearest city with a minimum of 10,000 inhabitants as a proxy for access to markets.

Summary statistics for all of the variables used in the regressions are reported in Table 1.

**Table 1:** Summary statistics for variables used in regressions.

**Dependent Variables (# of days per week consumed)**	**Mean**	**Std dev**
Animal source foods	3.87	2.85
Vitamin A rich fruits	1.66	2.21
Vitamin A rich vegs	1.21	1.94
Green leafy veg	3.77	2.78
‘Other’ fruits and veg	2.16	2.32
Legumes	2.01	2.55
**Independent Variables**		
Forest in ha	891	1,583
Timber Plantations in ha	272	910
Agr Crop Plantation in ha	548	1,323
Swidden/agroforest in ha	2,061	2,238
Father’s education	7.3	3.7
Wealth score	-58,710	80,112
Currently breastfed	0.61	0.49
Month Index	7.7	4.9
Elevation	194	312
Aridity index	15,102	3,894
Distance to city (in degrees)	0.43	0.42
Distance to coast (in degrees)	0.3`	0.35
Distance to river (in km)	3.7	5.8
Age in months	28	11
Muslim (yes = 1)	0.82	0.39
Male (yes = 1)	0.53	0.50
Observations	3,064	

### Regression Model

#### National Model

The following is our basic regression model:

$$F_{i}=\;\alpha +\beta A+\delta I+\theta H+\vartheta C+\;\sigma D+\mu P+\;\varepsilon$$

where *F* represents the frequency of consumption during the last seven days of a given food group and *i* is our index for food groups which includes: meat, green leafy vegetables, vitamin A rich fruits, vitamin A rich vegetables, ‘other’ fruits and vegetables. *A* is a vector of land area in various tree-dominated land use classes within 5 km of the child’s community with a baseline of all other land use classes. *I* is a vector of the child’s characteristics including age in months, age squared, sex, and a dummy for whether the child is currently breastfeeding. *H* is a vector of household characteristics including: wealth (based on an asset index), the number of years of the father’s education and a dummy equal to one if the household is Muslim. *C* is a vector of community characteristics including percentage of distance to the nearest city, distance to coast, distance to nearest river, aridity, and elevation. *D* is an ordered variable representing the month the interview took place to account for seasonal variations in dietary patterns. And *P* is a vector of province dummy variables that were included in the survey.

Since the dependent variable in our models, consumption frequency, is a ‘count’ variable, we run poisson or negative binomial regressions with standard errors clustered at the community level (poisson models were used when the variance was less than the mean and negative binomial regressions when the variance exceeded the mean). The main independent variables of interest are the vegetation classes characterized by tree cover.

#### Regional case studies

Indonesia is a vast archipelago with enormous diversity in geography, ecology, agricultural practices, and culture across regions. We include dummies for the 25 provinces found in the data in the overall models, in order to control for regional-level variations. Nevertheless, an aggregate analysis still combines effects that likely differ across regions. If relationships differ substantially in direction and magnitude across the sample, then a model run on the entire sample will be unable to uncover these effects. We therefore, also run the models for a sub-set of regions individually.

We choose four provinces–- Central Sulawesi, West Kalimantan, Central Java, and Riau—each of which is chosen to represent one of the tree-dominated land classes. Central Sulawesi is the province in the data set that has the largest area of natural forests. Kalimantan is the area in Indonesia most known for the practice of swidden cultivation [65, 66] and our data set indicates that West Kalimantan is the province in Kalimantan with the most area in the land class defined as ‘swidden/agroforests’ based on the MOF data from 2003. Central Java has the most land area with planted forests in the DHS dataset and Riau has the most land with agricultural tree crop plantations.

## Results

### National Model

Table 2 shows the regression coefficients and z-statistics from the aggregate model. The key variables of interest are the land class variables; therefore their coefficients are the focus of the discussion. We find no statistically significant relationship between area of natural forest and frequency of consumption of any of the six food groups. Children living in areas with more land in timber plantations eat both vitamin A rich fruit and legumes more frequently than children living in other areas. Children living in areas with more land used for tree crop plantations only eat legumes more frequently than children living in other areas. There is also a statistically significant positive relationship between area in swidden and frequency of legume consumption.

**Table 2:** All Indonesia (poisson and negative binomial regression results with standard errors clustered at DHS cluster level reported in parentheses).

	(1)	(2)	(3)	(4)	(5)	(6)
Independent Variables	Animal source foods	Vit A rich fruit	Vit A rich veg	Green veg	‘other’ fruit & veg	legumes
Forest area	2.35e-06	-8.52e-06	3.51e-05	-1.03e-05	-1.03e-05	1.57e-07
	(0.183)	(-0.304)	(0.953)	(-0.906)	(-0.906)	(0.00519)
Timber plantation area	1.47e-05	8.93e-05***	3.48e-05	5.70e-06	5.70e-06	6.47e-05**
	(0.862)	(2.951)	(0.864)	(0.316)	(0.316)	(2.163)
Agr Plantation area	5.08e-06	2.37e-05	3.78e-05	2.13e-06	2.13e-06	8.21e-05***
	(0.376)	(0.864)	(1.332)	(0.175)	(0.175)	(3.498)
Swidden/agroforest	-6.21e-06	-1.20e-05	-3.42e-06	-7.67e-06	-7.67e-06	3.04e-05*
	(-0.700)	(-0.763)	(-0.158)	(-0.998)	(-0.998)	(1.824)
Father’s education	0.0195***	0.0380***	0.0274***	0.0118***	0.0190***	0.0169**
	(4.776)	(4.730)	(2.726)	(2.693)	(2.836)	(2.157)
Wealth index	9.96e-07***	6.49e-07	2.97e-06***	-1.22e-07	3.40e-07	8.03e-07*
	(4.509)	(1.541)	(5.187)	(-0.552)	(1.034)	(1.934)
breastfeeding	-0.236***	-0.326***	-0.202***	-0.298***	-0.201***	-0.204***
	(-7.450)	(-5.758)	(-2.838)	(-10.03)	(-4.460)	(-3.939)
Month of survey	-0.00934**	0.00955	0.00429	0.00422	0.00503	0.000320
	(-2.453)	(1.319)	(0.465)	(1.151)	(0.897)	(0.0446)
Elevation	-1.77e-06	-2.71e-05	1.23e-05	4.42e-05	-1.55e-05	7.01e-06
	(-0.0290)	(-0.226)	(0.0812)	(0.842)	(-0.172)	(0.0572)
Aridity index	2.55e-07	-5.38e-06	-7.84e-06	-3.31e-07	5.61e-06	-2.31e-05*
	(0.0378)	(-0.425)	(-0.463)	(-0.0522)	(0.613)	(-1.885)
Distance to coast	-0.149**	0.244**	0.0621	0.0391	-0.0710	0.0275
	(-2.037)	(1.973)	(0.426)	(0.673)	(-0.752)	(0.225)
Distance to river	-2.00e-07	-8.42e-06	-6.47e-06	1.15e-06	6.38e-06	4.10e-07
	(-0.0849)	(-1.587)	(-0.631)	(0.463)	(1.070)	(0.0587)
Distance to city	0.0335	-0.130	-0.298**	-0.0701	-0.111	-0.169
	(0.550)	(-1.189)	(-2.111)	(-1.469)	(-1.407)	(-1.240)
Age in months	-0.000506	0.00753	-0.00884	-0.0130*	-0.00934	0.0131
	(-0.0711)	(0.603)	(-0.562)	(-1.919)	(-1.001)	(1.131)
Age squared	-9.27e-05	-0.000224	-4.15e-05	7.22e-05	8.66e-05	-0.000379**
	(-0.848)	(-1.156)	(-0.163)	(0.691)	(0.625)	(-2.146)
Muslim	0.0654	0.223*	-0.124	0.0127	0.0850	0.318**
	(1.001)	(1.727)	(-0.752)	(0.246)	(0.850)	(2.192)
Male	0.0311	-0.0291	0.00847	0.00299	0.00665	-0.0449
	(1.165)	(-0.591)	(0.268)	(0.109)	(0.157)	(-1.055)
Province dummies	yes	yes	yes	yes	yes	yes
Constant	1.333***	0.166	0.790*	1.496***	.7693***	1.124***
	(7.293)	(0.524)	(1.694)	(8.280)	(2.92)	(3.374)
Observations	3,063	3,044	3,082	3,064	3,050	3,039

Robust z-statistics in parentheses

*** p<0.01

** p<0.05

* p<0.1

As can be seen in Fig 1, there are large differences in the types of land use and cover across the archipelago. There are also vast differences in culture, infrastructure, and history. It is quite possible that the relationships between the different land use classes and diet also differ across the country and thus may be ‘hidden’ in the aggregate analysis. We, therefore, also run the models on four regional sub-samples.

### Four Provinces

Table 3 below summarizes the results for each land class category for each food group. We report the results only for the province that was chosen to represent the particular land class. Not all provinces have much land in some of the classes so it might be misleading to focus for example on results for ‘forests’ in a province which only has 100 ha of land in forests. The complete tables with all of the regression coefficients for each food group for each province can be found in S1–S4 Tables.

**Table 3:** Four Provinces (table indicates the sign of the coefficient when statistically significant and number of stars indicate statistical significance level *-90%; **-95%; ***-100%.

	Meat	VitaminA-rich fruit	VitaminA-rich Veg	Green leafy veg	‘Other’ fruit & veg	Legumes
Forests (Central Sulawesi)		* +	*** +	** +		
Timber Plantations (Central Java)	*+					
Agricultural Tree Crops (Riau)			*** +			
Swidden/Agroforestry (West Kalimantan)	** +	*** +		** +		*** +

We see that in Central Sulawesi, there is a positive relationship between the frequency of vitamin A rich fruit consumption, vitamin A rich vegetable consumption, and green vegetable consumption and the area around a community that is natural forest. In Central Java, there was a weak, but positive relationship between the area with timber plantations and frequency of consumption of meat. In Riau, children living in areas with more land cultivated with plantation crops only ate vitamin A rich vegetables more frequently than children with less land in that category. And finally, in West Kalimantan, children living in communities with more land under swidden/agroforests ate meat, vitamin A rich fruits, green vegetables, and legumes more frequently than children living in areas with less land under swidden/agroforests.

## Discussion

While it is useful to have national level aggregate studies that look for broad patterns in relationships, these can lead to very different results and conclusions than studies at a more local level, particularly in a country as geographically and culturally diverse as Indonesia. There are two reasons why this can occur. First, there may be unobserved variables that are correlated with the independent variables of interest that can affect the estimates. For example, land use practices may differ across ethnic groups as do cultural preferences for certain foods [54]. In a regression using the aggregate sample, certain places which are more likely to have different geographies and land uses are also more likely to have different ethnic groups with associated cultural preferences for foods. Second, there can be actual differences in the relationships between the land use practices themselves and diets in different regions due to such things as differences in soil quality or access rights and law enforcement. For example, access to forest land for swidden can differ across regions resulting in differences in soil quality and subsequent harvests and therefore diet. Similarly, law enforcement against illegal hunting may differ across regions affecting the relationships between the different land classes and frequency of consumption of animal source foods.

When we compare the results from our aggregate model as seen in Table 2 with the results from the provincial sub-samples in Table 3, we see that indeed, most of the provincial patterns are not maintained in the overall regression results. For example, in the overall sample, there is no statistically significant relationship between area of natural forest and consumption of any of the nutrient-rich food groups, while in forest-rich Central Sulawesi, children living in close proximity to forests consume micronutrient-rich foods more frequently than children living in other areas. There are any of number of reasons why this may be the case. For example, the forests in Central Sulawesi may have more fruit-bearing trees than in other regions; access to forests may be easier in the region due to different local laws and customs; local cultures may have greater knowledge of wild foods and/or value their taste more than in other regions.

Similarly, in the aggregate sample, there is a positive statistically significant relationship between frequency of consumption of vitamin-A rich fruits and legumes and the amount of land with timber plantations. But, when we focus only on Central Java—the province in the sample with the most land area used for timber plantations—there is no statistically significant relationship between area of timber plantation and the frequency of consumption of these foods, but there is a positive relationship between timber plantation area and meat consumption. In 2003, the five Javanese provinces had by far the majority of timber plantations in the sample. Thus it is possible that the timber variable picks up some of the characteristics of Java which are associated with more frequent fruit and legume consumption than in other islands. When we look at Central Java alone, there is no difference in consumption frequency of vitamin-A rich fruit and legumes in communities with or without timber plantations. We re-ran the aggregate model excluding the Javanese provinces from the analysis and indeed no longer find a statistically significant association between timber plantation area and frequency of consumption of vitamin-A rich fruit or legumes. This seems indicative that the timber plantation variable in the regressions using the aggregate sample is proxying for other regional effects.

Plantation crops such as palm oil, rubber, and coffee are often seen as important ways of reducing poverty and improving livelihoods in Indonesia. It is often assumed that better diets will follow from higher income. The aggregate model results show that children in areas with more land with plantation crops only eat legumes more frequently than children living in other areas. When we focus on the province of Riau (the province with the largest area of agricultural tree crop plantations) we see that children living in areas with more agricultural plantation crops eat vitamin-A rich vegetables with greater frequency than those with less land in these areas. Thus the Riau results are also different from those for the aggregate sample.

The aggregate sample also showed that only for legume consumption was there a statistically significant relationship between land area in swidden/agroforests, but no relationship was found for frequency of consumption of any of the other food groups. By contrast, in the West Kalimantan sub-sample, the results show a statistically significant relationship for frequency of consumption of animal source foods, vitamin-A rich fruits, and green vegetables as well as for legumes. This land class is difficult to interpret with certainty because it combines different types of land into one category; namely smallholder agroforestry activities and swidden cultivation. Thus for example, in areas where this represents agroforestry, the more frequent consumption of fruits that we see might be grown on planted trees, but in areas where it is swidden agriculture, these foods may be coming from planted crops, gathered from the wild, or from managed fallows. In many areas, both practices are combined as well [65, 67]. Swidden agriculture is often viewed as a backward and primitive practice that is an impediment to food security and contributes to deforestation and degradation [68,69]. If the land in the swidden/agroforest land use category for West Kalimantan is largely comprised of swidden lands, then both the Indonesian government and researchers will need to reconsider their views on its contribution to food security.

Any positive relationship between one of the land use categories and diet can be driven by a direct or indirect effect. The direct effect would be either through food grown or collected in the forests, fallows or fields. The indirect effects might work through purchases made as a result of income earned from plantations for example or through better access to foods as a result of improved infrastructure that might accompany large plantation development. We assume that the better diets found for children living in areas with more forests and more swidden/agroforestry are a result of direct effects since these tend to be relatively low remunerating activities. Indeed we find a weak positive association between land with timber plantations in Central Java and animal source food consumption, which is the food group most associated with income. Thus it is plausible that higher incomes in communities with timber plantations are used to purchase more animal source foods, but not more fruits and vegetables. The positive association between agricultural plantations in Riau and frequency of consumption of vitamin-A rich vegetables may be due to higher incomes or better market access as a result of improved infrastructure allowing purchase of these foods. It also might be a result of an omitted factor such as ethnicity. These plantations are well known to employ Javanese workers who may have different tastes in foods, rooted in a long culinary culture. If higher income is driving this result, it is somewhat surprising that we do not see the purchase of more animal source foods and vitamin-A rich fruits and ‘other’ fruits and vegetables as well.

## Conclusions

Much of the policy rhetoric on food security in Indonesia focuses on production of more rice since it is the main staple food (and thus source of calories) and also important for cultural, political and historical reasons [70]. Increased rice production however, does not address the widespread problem of micronutrient deficiency. Government run micronutrient supplementation programs and public-private partnerships encouraging fortification have been the primary policies targeted at reducing micronutrient deficiencies with little attention paid to dietary quality.

The results here suggest that the nature of the landscape and the agrarian context may affect dietary quality and such factors should be considered in both land use policies as well as those aimed at improving nutrition. Our results suggest that forests and tree-based systems could play an important role in supporting dietary quality in Indonesia, but further research is necessary for understanding the exact mechanisms and the regional differences in such relationships.

While the results for the aggregate data do not show strong associations between tree dominated land classes and diet, the provincial level analyses exhibit interesting relationships. We do not believe that these results are conclusive, but that they are suggestive enough of important relationships between forests, tree-based systems, and nutrition to warrant further investigation. The fact that the models run on the aggregate sample do not reflect the provincial level results underscores the importance of more targeted research at the local level across different regions of Indonesia. More detailed data on both quantities of food consumed as well as on the definitive land use from different regions is necessary before arriving at conclusions and policy recommendations.

Natural forest and semi-natural forest characterized by swidden/agroforestry were positively associated with a larger number of nutritionally important food groups in provinces dominated by these land uses, than timber and palm oil plantations were in the provinces dominated by these land uses. The swidden/agroforestry land use was associated with more frequent consumption of animal source foods which is a particularly important and limited food group for the poor in developing countries. These results together suggest that the nutritional benefits associated with commercial plantations may not be sufficient to compensate for the loss of previous benefits obtained from natural forests and swidden/agroforestry systems. Such a hypothesis however, can only be tested through detailed and rigorous investigation at local scales.

## Supporting Information

S1 Table Central Sulawesi regression results. (DOCX)

S2 Table Central Java regression results. (DOCX)

S3 Table Riau regression results. (DOCX)

S4 Table West Kalimantan regression results. (DOCX)
