# Supplementary material for: Forests, Trees, and Micronutrient-Rich Food Consumption in Indonesia
Source: PLoS One. 2016 May 17;11(5):e0154139. doi: 10.1371/journal.pone.0154139 (PMC4871346; doi:10.1371/journal.pone.0154139)
Supplement: S1 Table — (DOCX) [file pone.0154139.s001.docx]

**S1 Table: Central Sulawesi** (poisson and negative binomial regression results with standard errors clustered at DHS cluster level)

|  | (1) | (2) | (3) | (4) | (5) | (6) |
| --- | --- | --- | --- | --- | --- | --- |
|  |  |  |  |  |  |  |
| Independent Variables | Animal source foods | Vit A rich fruit | Vit A rich veg | Green veg | ‘other’ fruit & veg | legumes |
|  |  |  |  |  |  |  |
| Forest area | 6.89e-06 | .0002862* | .000381*** | .0001427** | 7.43e-05 | 0.000168 |
|  | (0.200) | (1.81) | (2.79) | (2.31) | (1.14) | (1.19) |
| Agr Plantation Crops | -6.48e-05*** | .0002561** | .000320*** | .0000782 | 1.96e-05 | 9.69e-05 |
|  | (-2.94) | (2.16) | (2.80) | (1.43) | (0.355) | (1.295) |
| Swidden/agroforest | -1.15e-05 | .0007179* | .001480** | .0007191** | 0.000722*** | .00106 |
|  | (-0.149) | (1.74) | (2.53) | (2.39) | (3.167) | (2.33) |
| Father’s education | -0.0204 | -0.0798* | -0.114*** | -0.00926 | -0.0555* | -0.0287 |
|  | (-1.205) | (-1.960) | (-2.680) | (-0.494) | (-1.691) | (-0.535) |
| Wealth index | 7.84e-07 | -1.58e-06 | 4.62e-06* | 1.91e-06 | 3.79e-06** | 3.44e-06 |
|  | (0.732) | (-0.680) | (1.838) | (1.300) | (1.979) | (0.848) |
| breastfeeding | -0.583*** | -0.0353 | -0.643** | -0.344* | -0.284 | -0.725** |
|  | (-4.067) | (-0.109) | (-2.508) | (-1.695) | (-1.100) | (-2.125) |
| Month of survey | -0.0116 | -0.124 | -0.258*** | -0.0996** | -0.148*** | -0.218** |
|  | (-0.862) | (-1.222) | (-3.348) | (-2.318) | (-3.148) | (-2.390) |
| Elevation | 0.00125*** | -0.00402 | -0.00445** | -0.00128** | -0.00143 | -0.00334 |
|  | (3.031) | (-1.357) | (-2.321) | (-2.337) | (-1.545) | (-1.306) |
| Aridity index | 8.14e-05** | -0.000146 | -0.000178 | -0.000124 | -0.000156 | -0.000166 |
|  | (2.100) | (-0.530) | (-0.929) | (-1.470) | (-1.355) | (-0.665) |
| Distance to coast | -1.654** | 3.322 | 4.632** | 0.990 | 1.516 | 1.871 |
|  | (-2.341) | (0.894) | (2.015) | (0.921) | (0.934) | (0.617) |
| Distance to river | -4.58e-06 | -.000213*** | -.0002205** | -.0000383 | -.0000919 | -.0001912 |
|  | (-0.17) | (-2.66) | (-1.73) | (-0.73) | (-1.35) | (-1.50) |
| Distance to city | -0.667** | 0.907 | 1.383 | 0.817 | 0.914 | 1.394 |
|  | (-2.202) | (0.523) | (1.384) | (1.400) | (1.157) | (0.997) |
| Age in months | -0.00195 | 0.223** | 0.353*** | -0.0356 | 0.0450 | 0.0124 |
|  | (-0.0699) | (2.116) | (4.351) | (-0.889) | (0.950) | (0.154) |
| Age squared | -0.000238 | -0.00431** | -0.00667*** | 0.000187 | -0.000885 | -0.000350 |
|  | (-0.599) | (-2.452) | (-3.988) | (0.322) | (-1.207) | (-0.287) |
| Muslim | -0.0613 | 1.354** | 1.036 | -0.347 | 0.646* | 0.226 |
|  | (-0.313) | (2.109) | (1.641) | (-1.477) | (1.885) | (0.420) |
| Male | -0.107 | -0.853*** | -1.075*** | -0.332* | -0.380 | -0.956*** |
|  | (-0.761) | (-3.091) | (-3.185) | (-1.866) | (-1.627) | (-5.100) |
| Constant | 2.054*** | -2.559 | -3.730 | 3.303*** | 1.979** | 2.196 |
|  | (3.282) | (-1.098) | (-1.518) | (3.214) | (2.036) | (1.063) |
|  |  |  |  |  |  |  |
| Observations | 123 | 123 | 123 | 123 | 123 | 123 |

Robust z-statistics in parentheses

*** p<0.01, ** p<0.05, * p<0.1
