# Supplementary material for: Forests, Trees, and Micronutrient-Rich Food Consumption in Indonesia
Source: PLoS One. 2016 May 17;11(5):e0154139. doi: 10.1371/journal.pone.0154139 (PMC4871346; doi:10.1371/journal.pone.0154139)
Supplement: S2 Table — (DOCX) [file pone.0154139.s002.docx]

**S2 Table: Central Java** (poisson and negative binomial regression results with standard errors clustered at DHS cluster level)

|  | (1) | (2) | (3) | (4) | (5) | (6) |
| --- | --- | --- | --- | --- | --- | --- |
| Independent Variables | Animal source foods | Vit A rich fruit | Vit A rich veg | Green veg | ‘other’ fruit & veg | legumes |
|  |  |  |  |  |  |  |
| Forest area | .00032** | -0.0002 | .0006* | 0.0001 | 0.0002 | 0.0004** |
|  | (2.17) | (-0.874) | (1.75) | (0.929) | (1.174) | (2.120) |
| Timber Plantations | .00004* | -5.92e-06 | 3.87e-08 | 1.13e-05 | -4.09e-05 | 4.17e-05 |
|  | (1.65) | (-0.10) | (0.000934) | (0.419) | (-0.983) | (1.279) |
| Agr Plantation Crops | -.0006*** | 0.0006** | -.001*** | -0.0006*** | -0.0008*** | -0.000320 |
|  | (-2.67) | (2.169) | (-3.264) | (-2.981) | (-2.950) | (-1.220) |
| Swidden/agroforest | 8.40e-06 | -6.18e-05 | 4.43e-06 | 8.47e-06 | -2.44e-05 | -4.46e-05 |
|  | (0.25) | (-1.157) | (0.0911) | (0.236) | (-0.480) | (-0.749) |
| Father’s education | 0.0803*** | 0.0516* | 0.0516* | 0.00949 | 0.0344 | 0.000647 |
|  | (3.653) | (1.656) | (1.908) | (0.464) | (1.593) | (0.0374) |
| Wealth index | -7.01e-07 | -3.02e-07 | 3.37e-06*** | -1.37e-07 | -2.74e-07 | -5.71e-07 |
|  | (-0.973) | (-0.242) | (2.946) | (-0.156) | (-0.209) | (-0.630) |
| breastfeeding | -0.155 | -0.401** | -0.241 | -0.273** | 0.265 | -0.209 |
|  | (-0.976) | (-2.024) | (-1.505) | (-2.073) | (1.607) | (-1.160) |
| Month of survey | 0.0167 | -0.0165 | -0.0208 | -0.0285** | 0.00918 | -0.0535*** |
|  | (1.394) | (-0.863) | (-1.226) | (-2.018) | (0.464) | (-3.364) |
| Elevation | 3.20e-05 | 0.000124 | 0.000658 | 1.48e-05 | -0.000118 | -0.000147 |
|  | (0.135) | (0.297) | (1.410) | (0.0618) | (-0.419) | (-0.420) |
| Aridity index | -1.60e-05 | 1.63e-05 | -0.000100*** | -1.47e-05 | 1.71e-05 | -4.35e-05* |
|  | (-0.889) | (0.464) | (-2.897) | (-0.698) | (0.543) | (-1.646) |
| Distance to coast | -0.480 | 0.272 | -0.861 | 0.390 | 0.0865 | -0.410 |
|  | (-1.539) | (0.428) | (-1.570) | (1.009) | (0.174) | (-0.935) |
| Distance to river | -.0000781 | .00006 | -.00009* | .00001 | -.00006 | -.00003 |
|  | (-1.56) | (0.74) | (-1.82) | (0.33) | (-0.95) | (-0.45) |
| Distance to city | -2.187** | -3.105 | 1.473 | -0.510 | 0.0672 | -0.0452 |
|  | (-2.472) | (-1.287) | (1.039) | (-0.539) | (0.0349) | (-0.0350) |
| Age in months | 0.0465 | 0.0217 | -0.0134 | -0.0555** | 0.0573 | 0.0760 |
|  | (0.966) | (0.325) | (-0.354) | (-2.213) | (1.595) | (1.543) |
| Age squared | -0.000815 | -0.000382 | 0.000144 | 0.000913*** | -0.000982* | -0.00159* |
|  | (-0.930) | (-0.305) | (0.216) | (2.600) | (-1.646) | (-1.666) |
| Muslim | 0.219 | -0.663*** | -0.0929 | -0.241 | -0.368 | -0.765*** |
|  | (0.543) | (-3.473) | (-0.219) | (-0.927) | (-1.520) | (-3.266) |
| Male | -0.118 | 0.0221 | 0.142 | 0.127 | -0.109 | -0.0969 |
|  | (-0.758) | (0.117) | (0.943) | (1.168) | (-0.586) | (-0.676) |
| Constant | 0.304 | 0.847 | 2.498*** | 2.516*** | -0.0665 | 2.575*** |
|  | (0.312) | (0.801) | (2.790) | (4.373) | (-0.0783) | (2.748) |
|  |  |  |  |  |  |  |
| Observations | 138 | 138 | 138 | 138 | 138 | 138 |

Robust z-statistics in parentheses

*** p<0.01, ** p<0.05, * p<0.1
