# Supplementary material for: Forests, Trees, and Micronutrient-Rich Food Consumption in Indonesia
Source: PLoS One. 2016 May 17;11(5):e0154139. doi: 10.1371/journal.pone.0154139 (PMC4871346; doi:10.1371/journal.pone.0154139)
Supplement: S3 Table — (DOCX) [file pone.0154139.s003.docx]

**S3 Table: Riau** (poisson and negative binomial regression results with standard errors clustered at DHS cluster level)

|  | (1) | (2) | (3) | (4) | (5) | (6) |
| --- | --- | --- | --- | --- | --- | --- |
| Independent Variables | Animal source foods | Vit A rich fruit | Vit A rich veg | Green veg | ‘other’ fruit & veg | legumes |
|  |  |  |  |  |  |  |
| Forest area | 5.11e-05 | -2.62e-05 | -0.000316*** | 2.05e-05 | -5.22e-05 | 0.000137 |
|  | (0.700) | (-0.152) | (-2.773) | (0.349) | (-0.294) | (1.114) |
| Timber crops | 0.00840 | 0.0397 | -0.00819 | 0.0208 | 0.00829 | 0.0179 |
|  | (0.531) | (0.908) | (-0.410) | (1.409) | (0.349) | (0.596) |
| Agr Plantation Crops | 0.000109 | 2.40e-06 | 0.000298*** | 1.20e-05 | 0.000114 | 0.000135 |
|  | (1.421) | (0.0267) | (3.066) | (0.231) | (1.408) | (0.939) |
| Swidden/agroforest | 9.57e-05 | -7.34e-05 | 0.000316** | 9.22e-05 | 0.000298*** | 0.000135 |
|  | (0.862) | (-0.611) | (2.434) | (1.271) | (2.694) | (0.720) |
| Father’s education | 0.0149 | 0.0392 | 0.0227 | 0.0253 | 0.0253 | 0.0303 |
|  | (0.781) | (0.649) | (0.392) | (1.094) | (0.642) | (0.908) |
| Wealth index | 1.24e-06 | 3.83e-06* | 9.54e-06*** | 1.06e-06 | 2.74e-06* | 1.67e-06 |
|  | (0.953) | (1.950) | (4.132) | (0.840) | (1.927) | (0.867) |
| breastfeeding | -0.171 | -0.325 | -0.184 | -0.297* | -0.174 | -0.300 |
|  | (-1.262) | (-1.011) | (-0.461) | (-1.652) | (-0.569) | (-0.984) |
| Month of survey | 0.00907 | 0.0702* | 0.0456* | 0.0194 | 0.0400 | -0.0507 |
|  | (0.375) | (1.729) | (1.811) | (1.049) | (1.463) | (-1.401) |
| Elevation | 0.000964 | 0.0147 | 0.0247*** | 0.00906 | 0.0131 | 0.0129 |
|  | (0.177) | (1.531) | (3.005) | (1.433) | (1.507) | (1.453) |
| Aridity index | 8.35e-05 | -9.81e-05 | -0.000808*** | -0.000326*** | -0.000142 | -1.91e-05 |
|  | (1.096) | (-0.579) | (-5.726) | (-3.393) | (-1.173) | (-0.136) |
| Distance to coast | -1.164** | -0.361 | -1.878*** | -0.592 | -1.734** | -0.676 |
|  | (-2.047) | (-0.520) | (-2.759) | (-1.205) | (-2.069) | (-0.747) |
| Distance to city | -0.134 | -0.201 | 0.586** | -0.207 | -0.163 | -0.394 |
|  | (-0.865) | (-0.626) | (2.247) | (-1.188) | (-1.092) | (-1.294) |
| Distance to river | 3.01e-05 | -2.53e-05 | -3.71e-05 | -3.88e-05 | -1.51e-05 | 1.31e-05 |
|  | (1.537) | (-0.720) | (-1.139) | (-1.393) | (-0.267) | (0.300) |
| Age in months | -0.0412 | -0.00409 | -0.173** | -0.0645* | -0.0402 | -0.0572 |
|  | (-1.272) | (-0.0528) | (-2.569) | (-1.715) | (-0.681) | (-0.798) |
| Age squared | 0.000512 | -7.13e-05 | 0.00229** | 0.000805 | 0.000450 | 0.000839 |
|  | (1.028) | (-0.0570) | (2.275) | (1.535) | (0.522) | (0.922) |
| Muslim | 0.652** | 0.825 | 3.378*** | -0.0495 | 15.24*** | 15.10*** |
|  | (2.190) | (0.815) | (5.018) | (-0.304) | (19.28) | (11.22) |
| Male | -0.0304 | -0.0665 | 0.599 | -0.297** | -0.363** | -0.384 |
|  | (-0.236) | (-0.240) | (1.613) | (-2.414) | (-1.986) | (-1.582) |
| Constant | 0.0465 | 1.131 | 10.85*** | 7.798*** | -11.68*** | -13.52*** |
|  | (0.0317) | (0.329) | (4.717) | (4.820) | (-6.492) | (-4.431) |
|  |  |  |  |  |  |  |
| Observations | 103 | 103 | 103 | 103 | 102 | 103 |

Robust z-statistics in parentheses

*** p<0.01, ** p<0.05, * p<0.1
