# Supplementary material for: Forests, Trees, and Micronutrient-Rich Food Consumption in Indonesia
Source: PLoS One. 2016 May 17;11(5):e0154139. doi: 10.1371/journal.pone.0154139 (PMC4871346; doi:10.1371/journal.pone.0154139)
Supplement: S4 Table — (DOCX) [file pone.0154139.s004.docx]

**S4 Table: West Kalimantan** (poisson and negative binomial regression results with standard errors clustered at DHS cluster level)

|  | (1) | (2) | (3) | (4) | (5) | (6) |
| --- | --- | --- | --- | --- | --- | --- |
| Independent Variables | Animal source foods | Vit A rich fruit | Vit A rich veg | Green veg | ‘other’ fruit & veg | legumes |
|  |  |  |  |  |  |  |
| Forest area | -5.64e-05 | -0.000175*** | -0.000118 | -6.92e-05 | -6.08e-05 | -9.97e-05 |
|  | (-0.917) | (-3.455) | (-1.115) | (-1.578) | (-0.859) | (-0.771) |
| Swidden/agroforest | 0.000143** | 0.000204*** | 0.000165 | 0.000127** | 8.44e-05 | 0.000378*** |
|  | (2.127) | (3.260) | (1.260) | (2.550) | (1.008) | (3.029) |
| Agr. Plantation Crops | 3.99e-05 | 6.79e-05 | 0.000132 | 3.73e-05 | 3.24e-05 | 0.000190 |
|  | (0.824) | (1.393) | (1.637) | (1.029) | (0.698) | (1.585) |
| Father’s education | -0.0194 | 0.0323 | 0.0230 | -0.0145 | 0.00469 | -0.0519 |
|  | (-1.061) | (1.214) | (0.585) | (-0.581) | (0.157) | (-1.351) |
| Wealth index | 1.02e-06 | -9.18e-07 | 3.37e-07 | 1.54e-06 | -1.14e-06 | 4.95e-06* |
|  | (0.879) | (-0.706) | (0.151) | (1.577) | (-0.559) | (1.799) |
| breastfeeding | -0.0885 | -0.0916 | -0.574** | -0.368*** | -0.139 | -0.194 |
|  | (-0.518) | (-0.501) | (-2.283) | (-3.026) | (-0.665) | (-0.591) |
| Month of survey | -0.00352 | -0.0338** | -0.00904 | 0.0222 | 0.00267 | -0.0738* |
|  | (-0.198) | (-2.294) | (-0.285) | (1.470) | (0.121) | (-1.801) |
| Elevation | -0.0157** | -0.0109 | -0.0172 | -0.0110* | -0.00597 | -0.0142 |
|  | (-2.059) | (-1.441) | (-1.285) | (-1.833) | (-0.667) | (-1.103) |
| Aridity index | -0.000136* | 5.73e-05 | -0.000152 | -6.76e-05 | 0.000137* | -0.000482** |
|  | (-1.782) | (0.590) | (-1.007) | (-0.971) | (1.875) | (-2.457) |
| Distance to coast | -0.675** | -0.948*** | 0.0997 | -0.249 | -0.828** | -1.257* |
|  | (-2.150) | (-3.044) | (0.132) | (-0.724) | (-2.318) | (-1.771) |
| Distance to river | 6.62e-05 | -3.51e-06 | 7.58e-05 | 6.99e-05** | 6.62e-05* | 6.74e-05 |
|  | (1.571) | (-0.106) | (1.339) | (2.562) | (1.906) | (0.772) |
| Distance to city | 0.403** | 0.284* | 0.135 | 0.106 | 0.182 | 0.806** |
|  | (1.979) | (1.782) | (0.408) | (0.642) | (1.052) | (2.035) |
| Age in months | 0.0587 | 0.0420 | -0.0441 | -0.00735 | 0.0101 | -0.00656 |
|  | (1.180) | (0.887) | (-0.790) | (-0.274) | (0.198) | (-0.113) |
| Age squared | -0.000951 | -0.000785 | 0.000473 | -5.39e-06 | -0.000191 | -0.000828 |
|  | (-1.088) | (-1.100) | (0.562) | (-0.0125) | (-0.248) | (-0.809) |
| Muslim | 0.0463 | 0.747*** | -0.299 | 0.268 | 0.203 | 0.548 |
|  | (0.227) | (3.051) | (-0.678) | (1.532) | (0.685) | (1.442) |
| Male | 0.0392 | -0.262 | -0.160 | -0.148 | -0.242 | 0.0248 |
|  | (0.302) | (-1.184) | (-0.749) | (-1.270) | (-1.199) | (0.101) |
| Constant | 2.667 | -1.434 | 3.877 | 2.412* | -2.292 | 9.706*** |
|  | (1.585) | (-1.217) | (1.427) | (1.794) | (-1.506) | (2.591) |
|  |  |  |  |  |  |  |
| Observations | 162 | 162 | 162 | 162 | 162 | 162 |

Robust z-statistics in parentheses

*** p<0.01, ** p<0.05, * p<0.1
